# Book Reviews

**Published:** 1902-03

**Authors:** 


INTERNATIONAL CLINICS. A Quarterly of Clinical Lectures and Especially Prepared Articles on Medicine, Neurology, Surgery, Therapeutics, Obstetrics, Pediatrics, Pathology, Dermatology, Diseases of the Eye, Ear, Nose and Throat, etc Edited by HENRY W. CATTELL, A. M., M. D., of Philadelphia, with the collaboration of John B. Murphy, M. D., of Chicago; Alexander D Blackader, M. D., of Montreal; H. C. Wood, M. D., of Philadelphia; T. M. Rotch, M. D , of Boston; E. Landolt, M. D., of Paris; Thomas G. Morton, M. D., and Charles H Reed, M. D., of Philadelphia; J. W. Ballantyne, M. D., of Edinburgh, and John Harold, M. D., of London. Volume III. Eleventh series. Philadelphia: J. B. Lippincott Co. 1901. [Price, cloth, $2.00 each; half-leather, $2.25 each.]

This volume contains lectures upon therapeutics, medicine, neurology, surgery, and diseases of the eye and throat, together with a paper on laboratory methods, the latter by C. N. B. Camac, of New York. Among the foreign contributors are John Abercrombie, William Henry Battle, J. W. H. Eyre, Amand Routh and A. H. Tubby, of London; Valdemar Bie, of Copenhagen; Patrick J. Fagan, of Dublin; Alexander James, of Edinburgh; Louis Jullien, Edmund Landolt, Paul Reclus and A. Routier, of Paris; Augusto Murri, of Bologna; Arnold Pick, of Prague, and Demetrius Roncali, of Rome. Among the domestic contributors are D. R. Brower, W. Cheatham, Solomon Solis-Cohen, T. D. Crothers, John B. Deaver, George M. Edebohls, Beverly Robinson and Charles F. Withington. Some of the lectures are illustrated with half-tones and drawings. The standard of this excellent series is well maintained in this volume.

THE STANDARD MEDICAL DIRECTORY OF NORTH AMERICA. Consisting of Twelve Parts, including Directory of Physicians of North America, Medical Colleges, Medical Service of the United States, Medical Societies, Medical Practice Acts, Medical Publications (including Books and Periodicals), Mineral Springs, Drugs and Medicines, Medical and Surgical Products, Manufacturers and Life Insurance Companies. Chicago: G. P. Engelhard & Co. 1902. [Price, $10 00.]

This directory is a handsome imperial octavo book of 925 pages, printed in clear type with the names of places in heavy face, and divided into sections, the latter numbering twelve parts. This arrangement facilitates reference and the general plan is such as to exhibit in concise form the various interests represented in the respective parts.

Such a book involves an enormous outlay of money and it is surprising, considering the difficulties encountered, that such accuracy of detail has been attained. One of the interesting features of the book is part 5, entitled medical legislation which gives the text of the medical practice acts of all the states; a synopsis of the statutes and of board regulations and rulings, together with the personnel of the state boards of health, and in most instances of the state boards of medical examiners. In the latter respect, however, some of the states have been inadequately reported, notably Alabama, Connecticut, New York and Pennsylvania.

The book is substantially bound in red buckram, decorated in gold, and is a credit in every way to the publishers.

---

PROGRESSIVE MEDICINE. Vol. IV., 1901. A Quarterly Digest of Advances, Discoveries and Improvements in the Medical and Surgical Sciences. Edited by HOBART AMORY HARE, M. D., Professor of Therapeutics and Materia Medica in the Jefferson Medical College of Philadelphia. Octavo, handsomely bound in cloth, 400 pages, 13 illustrations. Philadelphia and New York: Lea Brothers & Co. [Per annum, in four cloth-bound volumes, $10.00.]

The contents of this volume include diseases of the digestive tract and allied organs, the liver, pancreas and peritoneum, by Max Einhorn; genito-urinary diseases, by William T. Belfield; anesthetics, fractures, dislocations, amputations, surgery of the extremities and orthopedics, by Joseph C. Boodgood; diseases of the kidneys, by John Rose Bradford; physiology, by Albert Brubaker; hygiene, by Henry B. Baker; practical therapeutic referendum, by E. Q. Thornton.

In each of these sections, all of which have been prepared by men of experience, recent advances of value are recorded in such a way as to make them of practical value to the everyday practitioner of medicine, whether generalist or specialist. The reader will readily observe that it contains a series of critical reviews by men who are distinguished in medicine. The four volumes for 1901 afford a vast amount of valuable material that cannot be found elsewhere.

A TEXT-BOOK OF OBSTETRICS. By BARTON COOKE HIRST, M. D., Professor of Obstetrics in the University of Pennsylvania. Third edition, thoroughly revised and enlarged. Royal 8vo, 873 pages, with 704 illustrations, many of them in colors. Philadelphia and London: W. B. Saunders & Co. 1901. [Cloth, $5.00 net.]

The third edition of this book does not differ from the previous editions except in the following particulars: The chapters on diagnosis of pregnancy, the pathology of pregnancy, the pathology of labor, and obstetrical operations, contain considerable new material, and many other chapters have been revised in some respects; over fifty new illustrations, including three colored plates have been added, most of which are original. The size of the book, however, remains as before owing to condensation of the text or elimination, as the case may be.

It is a text-book that represents the present status of the obstetric art, is written by an experienced teacher, and altogether possesses many attractions for both pupil and teacher, to whom it is heartily recommended.

